# Towards evidence-based response criteria for cancer immunotherapy

**DOI:** 10.1038/s41467-023-38837-3

**Published:** 2023-05-24

**Authors:** Elena Garralda, Scott A. Laurie, Lesley Seymour, Elisabeth G. E. de Vries

**Affiliations:** 1 Research Unit, Vall d’Hebron Institute of Oncology, Barcelona, Spain; 2 Division of Medical Oncology, The Ottawa Hospital Cancer Centre, Ottawa, Canada; 3 Canadian Cancer Trials Group, Queens University, Cancer Centre of South Eastern Ontario, Kingston, ON Canada; 4 Department of Medical Oncology, University Medical Center Groningen, University of Groningen, Groningen, the Netherlands

**Keywords:** Tumour immunology, Cancer imaging, Tumour immunology

## Abstract

Early detection of immunotherapy-induced tumor response is of major benefit for patients but can be complicated by therapy-induced pseudoprogression. A consensus guideline-iRECIST- was developed as a modification of Response Evaluation Criteria in Solid Tumours (RECIST version 1.1). Here we describe which next steps are required to test its validity and how novel approaches for response criteria might be developed and included.

While a tremendous advance in oncology was achieved with the introduction of immune checkpoint inhibitors for several tumor types, the end goal has not been reached, as many patients still do not respond to immunotherapy. Numerous new immunotherapeutic treatments are being developed and are also being combined with other drugs^1^. Currently, there are an incredible number of studies underway. In 2022 there were 4897 active clinical trials reported testing anti-programmed cell death protein 1 (PD1)/programmed death-ligand 1 (PD-L1) monoclonal antibodies – as monotherapy or in combination with other treatments^2^. Additionally, many studies are ongoing with other immune-based treatments, including modified antibodies and cell therapies. According to data retrieved from ClinicalTrials.gov, as of April 2022, there were 1800 active cell therapy trials, most in hematological malignancies and 43% for solid tumors^3^, and in 2022 a total of 443 new cell therapy trials were initiated^4^.

In any drug development process, but especially with such a large number of potentially toxic agents in development, it is critically important to be able to decide early whether the novel treatment approach is active and, thus, relevant and worth further study. In this early stage, surrogate endpoints for assessment of antitumor effect in the metastatic disease often are used, namely objective tumor response rate and progression-free survival. Between 2017 and 2021 most of the Food and Drug Administration (FDA), namely 146 approvals of anti-cancer agents in the advanced-stage disease setting, did not have direct evidence of improved overall survival or quality of life. They used surrogate outcome data, including overall response rate and duration of response in 58 single-arm trials or progression-free survival in 39 randomized clinical trials^5^.

These response-based surrogate endpoints are based on the Response Evaluation Criteria in Solid Tumours (RECIST) criteria published in 2000 as a simplified but validated endpoint^6^. An updated version (1.1) appeared in 2009 after further validation on an extensive database, also including industry contributions, comprising information on over 6000 patients, illustrating the importance of data sharing^7^. With the availability of targeted agents, it was suggested that these criteria, validated on chemotherapy datasets, may not be applicable to these agents. Therefore, the value of RECIST v1.1 was tested after having obtained data from 23,259 patients with cancer, of whom 15,620 received targeted agents^8^. Landmark analyses showed an ordinal relationship between percentage change in tumor size from baseline to 12 weeks and overall survival, reinforcing that RECIST v1.1 performs well for response assessment of targeted agents (Fig. 1).

**Fig. 1:**
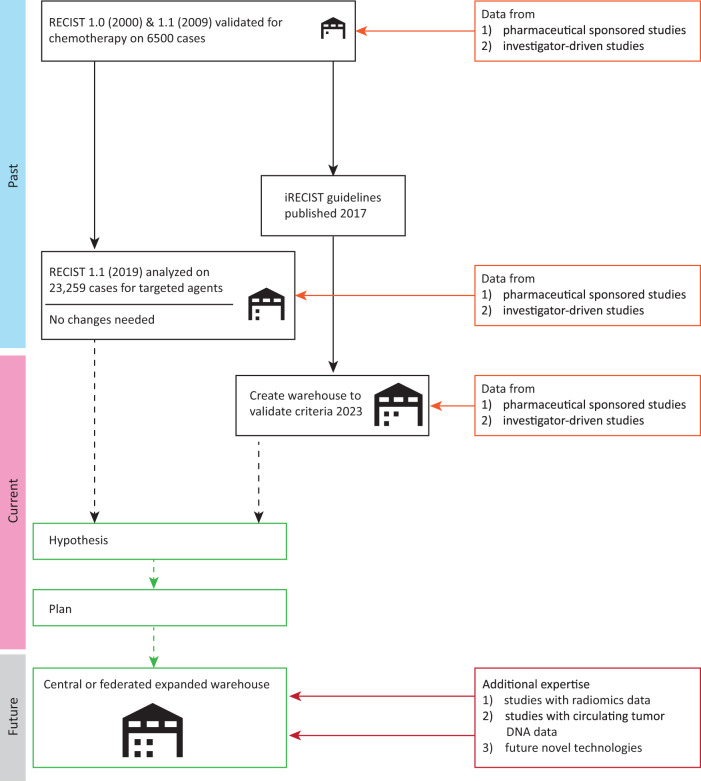
Evolution of RECIST. This figure shows the activities regarding the development of RECIST in the past (in blue), currently (in pink) and to be expected in the future (in gray). The increasing size of the warehouse represents the increasing number of data in the warehouse.

But the clinical application of immunotherapy raised a new issue. Patients treated with immunotherapy can develop a temporary ‘pseudoprogression’, which is an increase in the size of lesions, or the visualization of new lesions, followed by stable disease or a response. This could cause premature treatment withdrawal when interpreted as progressive disease according to RECIST criteria. Therefore in 2017, a consensus guideline-iRECIST- was developed by the RECIST working group for use in cancer immunotherapy trials^9^. The primary aim was to ensure consistent design and data collection, facilitate the ongoing collection of trial data, and ultimate validation of the guideline. iRECIST describes a standard approach to solid tumor measurements and definitions for objective change in tumor size for use in trials in which immunotherapy is used and allows continued treatment with the first assessment that meets iRECIST definitions of progression, providing the patient does not have a deteriorating condition until the progressive disease is confirmed at a subsequent response assessment. Five years after implementation, the RECIST committee continues to try to collect data from immunotherapy studies and studies with immunotherapy plus targeted therapy or chemotherapy trials as well as their standard treatment arms, to validate iRECIST, to create a warehouse to define the optimal RECIST criteria for immunotherapeutic agents.

In order to develop, refine and validate endpoints such as response and iRECIST, data sharing is critical but remains a current major challenge^10,11^. It requires contributions of data from investigator drives studies as well as from studies performed by pharmaceutical companies.

It is important to recognize that the validation of response guidelines is agnostic and only requires the use of data acquired in clinical trials with immunotherapeutic medicines and does not re-evaluate efficacy in the specific trial of a specific treatment or compare outcomes between agents. Instead, the pooled data are used to test how changes in tumor lesions correlate with overall survival for patients receiving immunotherapy.

One may wonder if validating iRECIST remains an important endeavor after five years of the initial publication. Currently, most, if not all, of the immunotherapy clinical trials allow continued treatment beyond RECIST v1.1 progressive disease, and most include, in addition to RECIST v1.1, also iRECIST or other versions of immune response guidelines. While we do not foresee that the capacity to continue treatment beyond progression will change or needs to change, it would be essential to validate iRECIST in order to evaluate the need to collect these further measures in the electronic case record form (eCRF), as well as its value as a surrogate measure of response (Fig. 1). With the number of clinical trials performed during the last five years with immunotherapy agents, validating iRECIST is within our hands and remains our responsibility as a scientific community and stakeholders in drug development to pursue this.

Moreover, the development of innovative imaging techniques, including positron emission tomography (PET) imaging with radiolabeled immune checkpoint inhibitors and tracers targeting CD8, is ongoing to improve response assessment^12^. The most developed are analyses of CT and magnetic resonance to extract more features and study whether such a radiomics approach could enrich the information for response analyses. One study applied radiomics and machine learning to analyze CT images obtained at baseline and first follow-up and their associated clinical metadata in patients with advanced melanoma on KEYNOTE-002 (pembrolizumab versus chemotherapy in participants) and KEYNOTE-006 (evaluation of safety and efficacy of two different dosing schedules of pembrolizumab compared to ipilimumab)^13^. Findings suggested that the radiomic signature discerned from CT images at baseline and on first follow-up may be used as an accurate early readout of future overall survival probability in these patients treated with single-agent anti-PD-1 antibody.

However, imaging does not have to be the only way to determine tumor response. In this respect circulating tumor DNA has drawn a lot of attention. A systematic review, in which 18 trials were included, was performed to study changes in circulating tumor DNA and outcomes in solid tumors treated with immune checkpoint inhibitors^14^. It was concluded that in advanced solid tumors, a reduction in circulating tumor DNA levels in response to immune checkpoint inhibitors is associated with substantial improvements in outcome. Circulating tumor DNA change is an early response biomarker that may allow for a reduced frequency of cross-sectional imaging in patients receiving immune checkpoint inhibitors^12^.

The RECIST working group has held international, multidisciplinary meetings to further evaluate the potential role of radiomics and circulating tumor DNA in the evaluation of response, and not only in the case of immunotherapy, as a surrogate endpoint (Fig. 1). Currently, specific manuscripts to address the challenges and describe the potential pathway to implement them within RECIST response evaluation are underway (Fig. 1). One of the main strengths of RECIST is its broad applicability and scalability through different tumor types, across clinical trials, and across centers in clinical trials. However, tumor types where standard response criteria have limitations, have tumor-specific response criteria, such as the inclusion of bone scans^15^ for prostate cancer with often bone disease. Even if not broadly incorporated, one could envision the addition of emerging technologies in a subset of patients or in situations where RECIST has limitations. Once again, for these or other new technologies to be incorporated into response criteria in a validated manner, encouraging data sharing is important.

## Conclusions

RECIST has become the widely adopted standard criteria used in early clinical trials of novel agents for solid tumors to determine the response-based endpoints, response rate, duration of response, and progression-free survival. Its validity has been confirmed for cytotoxic chemotherapy and for targeted therapy using shared data on patients enrolled in both academic and industry-sponsored clinical trials. Proposed criteria, iRECIST, have been incorporated as an exploratory endpoint into many clinical trials of immunotherapeutic agents in order to capture unusual patterns of response that can occur with these agents. Validation of iRECIST will require sharing clinical trial data from patients receiving immunotherapy agents. These analyses will look at the data in aggregate, without regard to the specific agent used, and will not compare agents. The analyses will be performed simply to confirm the usefulness of iRECIST as a surrogate endpoint in trials of immunotherapy agents and to see if iRECIST adds more than standard RECIST criteria in this situation. If so, then iRECIST could be adopted as the standard response assessment for immunotherapeutic agents. It can be envisioned that novel imaging and imaging analyses, as well as circulating tumor DNA approaches for response criteria, might be developed and included in the future.
